# A Case of Acute Eosinophilic Pneumonia Triggered by the SARS-CoV-2 Virus

**DOI:** 10.7759/cureus.38111

**Published:** 2023-04-25

**Authors:** Inês De Albuquerque Monteiro, Pedro Fernandes Moura, Diana Fernandes, José Carlos Carneiro, Sofia Teixeira

**Affiliations:** 1 Internal Medicine, Centro Hospitalar do Médio Ave, Vila Nova de Famalicão, PRT; 2 Pulmonology, Centro Hospitalar do Médio Ave, Vila Nova de Famalicão, PRT

**Keywords:** eosinophilic pneumonia, pulmonary consolidation, eosinophilia pneumonia, covid-19, sars-cov-2, eosinophilia

## Abstract

We report a case of acute eosinophilic pneumonia (AEP) triggered by the coronavirus disease 2019 (COVID-19) infection. A 60-year-old male with chronic sinusitis and tobacco use presented to the emergency department (ED) with an acute onset of dyspnea, non-productive cough, and fever. A diagnosis of moderate SARS-CoV-2 infection with bacterial superinfection was made. He was discharged on antibiotic therapy. One month later, due to the persistence of symptoms, he returned to the ED. At this time, blood analysis showed eosinophilia and a chest computed tomography scan showed bilateral diffuse infiltrative changes. He was admitted to the hospital for the study of eosinophilic disease. A lung biopsy was performed, which showed eosinophilic pneumonia. Corticotherapy was started with symptoms and peripheral eosinophilia resolution, and imaging improvement.

## Introduction

Acute eosinophilic pneumonia (AEP) is an acute rare lung disease characterized by eosinophilic infiltrates associated with smoking habits, inhalational exposures, drugs, viral infections, or idiopathic etiology [1,2]. It presents with acute febrile illness of less than one month, respiratory failure, consolidation, and diffuse ground-glass opacities on chest computed tomography (CT), bronchoalveolar lavage (BAL) fluid with eosinophilia >25% and/or infiltrations of eosinophils in the lung parenchyma on lung biopsy, and absence of known causes [1,3]. Severity can go from mild disease to acute respiratory distress syndrome, with potential progression to death.

This article was previously presented as a poster at the 20th European Congress of Internal Medicine 2022 and as a meeting abstract in the Abstract Book of the 20th European Congress of Internal Medicine.

## Case presentation

We present a 60-year-old male patient with chronic sinusitis and tobacco use who presented to the emergency department with an acute onset of dyspnea, non-productive cough, and fever. From the study carried out, leukocytosis at 11,500/uL (normal: 4000-10,000/uL) was highlighted, without other changes in the formula, with C-reactive protein at 12 mg/dL (normal: <1 mg/dL) and procalcitonin at 1.2 ng/mL (normal: <0.05 ng/mL). Thorax X-ray was suggestive of peripheral consolidation. The diagnosis of coronavirus disease 2019 (COVID-19) was confirmed by reverse transcription-polymerase chain reaction (rt-PCR). The patient was discharged with a diagnosis of COVID-19 infection, with bacterial superinfection assumed based on the procalcitonin value and persistent fever, cough, and dyspnoea, medicated with amoxicillin/clavulanic acid.

**Figure 1:**
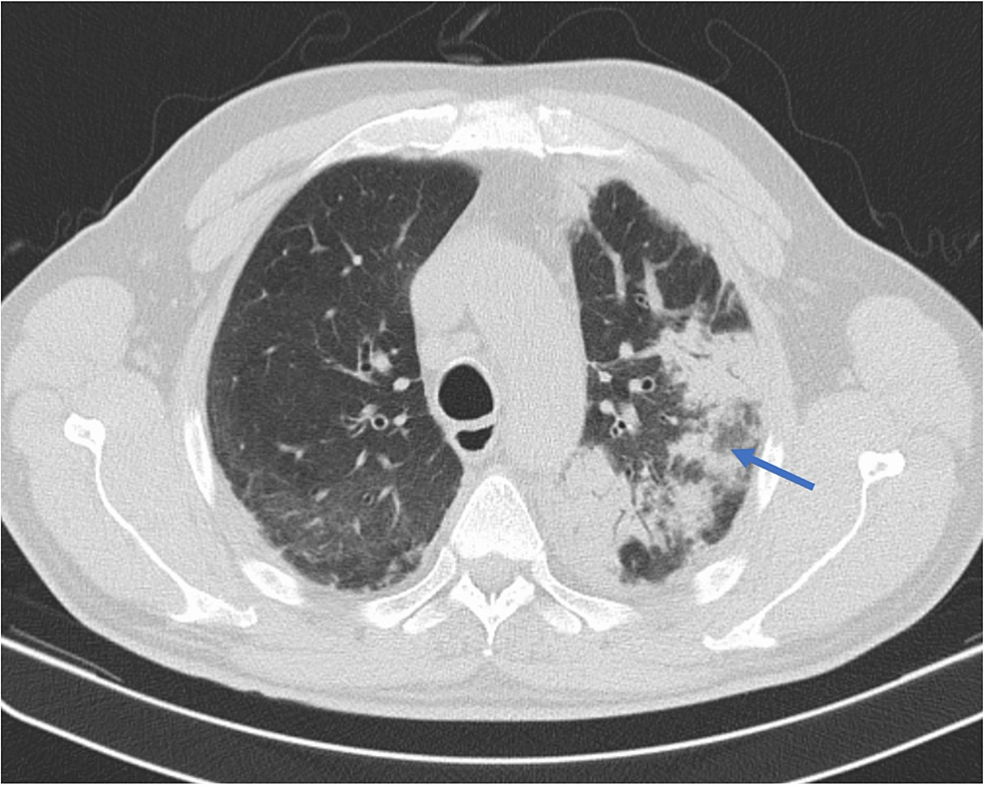
Admission CT showing consolidation of the lung parenchyma

He came back to the hospital one month later with persistent dyspnea, cough, and fever. Analytically, the following findings stand out: leukocytes at 12,860/UL (normal: 4000-10,000/uL), lymphocytes at 1520/uL (normal: 900-4000/uL), eosinophilia at 3000/uL (normal: 40-500/uL), and elevation of the C-reactive protein (16.58 mg/dL; normal: <1 mg/dL). Chest CT identified areas of consolidation of the lung parenchyma bilaterally, particularly in the apical segments (Figure 1). He was admitted to the internal medicine ward to proceed study for AEP.

An extensive etiological study was performed, which ruled out passed infection/passed immunity for cytomegalovirus (CMV) and hepatitis B virus (HBV), with no HIV or hepatitis C virus (HCV) infection. Anti-syphilis antibodies were negative, with negative micro and mycobiological blood cultures, as well as negative urine cultures and respiratory panels. An autoimmune study was negative for antinuclear antibody (ANA), antineutrophil cytoplasmic antibody (ANCA), and double-stranded DNA antibodies (ds-DNA). IgG, IgA, IgM, total immunoglobulin E (IgE), and serum angiotensin-converting enzyme results were in the normal range, with no consumption of complement and high erythrocyte sedimentation rate (37 mm/hr) (normal: <20 mm/hr). To confirm the diagnosis, BAL and lung biopsy were performed. BAL was negative for neoplastic cells and fungi, without other microbiological isolates, and research for SARS-CoV-2 was positive. It also showed increased cellularity with eosinophilia (54%) and normal lymphocyte and neutrophil counts. A lung biopsy was also performed and showed lung parenchyma with alveolar spaces filled with eosinophils and macrophages associated with inflammatory infiltrate in the alveolar septa. With these results, the diagnosis of AEP was established and prednisolone 75 mg/day (1 mg/kg/day) was initiated. A gradual resolution of symptoms and a marked decrease in peripheral eosinophilia (100/uL; normal: 40-500/uL) and improvement in imaging tests occurred. He was discharged after five days of corticosteroid therapy, with advice for pulmonology and internal medicine consultation.

**Figure 2:**
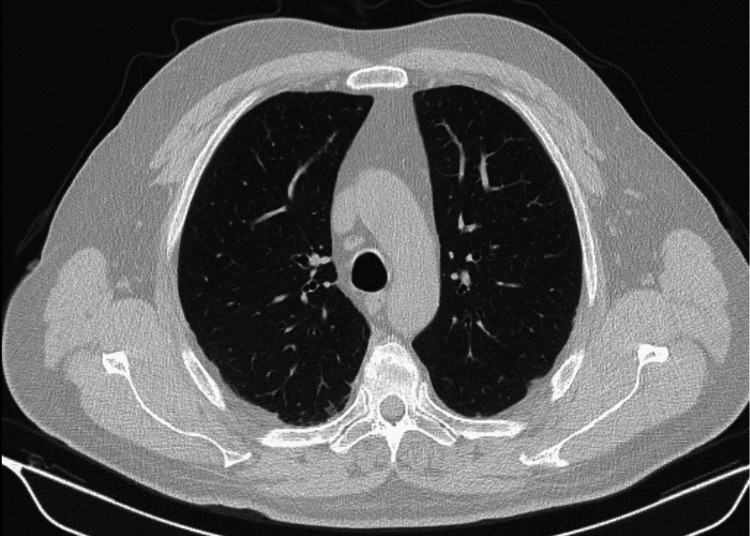
Chest CT reevaluation five months after diagnosis and initiation of corticosteroid therapy

Two weeks later, he was reassessed, and since he maintained clinical and analytical stability, the corticosteroid dose was reduced. Four months later and after progressive reduction of corticosteroids, the weaning regimen was started, being reassessed in the fifth month with a new chest CT (Figure 2) and blood work with peripheral eosinophilia (70/uL; normal: 40-500/uL). On imaging reassessment, there were no lesions in the lung parenchyma, with a resolved pattern of consolidation. During this period, he developed iatrogenic diabetes mellitus by corticosteroids and started metformin 500 mg twice a day, with good glycemic control.

One year after diagnosis, the patient was already without corticotherapy with no analytical or imaging recurrence of the disease.

## Discussion

AEP is a rare disease characterized by eosinophilic infiltrates in the lung parenchyma, which may be idiopathic or secondary to several agents. Initially, AEP was described as an acute respiratory disease of unidentified cause, but recently, tobacco, drugs, and infections were identified as etiologic factors [1,2]. There are several complications, especially at the pulmonary level, which have been described since the emergence of the COVID-19 pandemic. Since AEP can be caused by infections, we cannot rule out the possibility that the SARS-CoV-2 infection also has this evolution. Three previous studies describe AEP after SARS-CoV-2 infection, raising the hypothesis that it is a predisposing factor for the development of the disease [3-5]. The usual treatment for AEP involves the administration of corticosteroids in high doses to alleviate inflammation and inhibit the immune system. Generally, patients with AEP respond positively to this treatment and achieve a full recovery, although a few may suffer from lasting lung damage.

Our patient maintained smoking habits but was not exposed to toxins or illicit drugs, and was not taking any medication. He showed clear clinical, analytical, and imaging improvement, remaining stable after a one-year follow-up.

## Conclusions

Although reported cases of AEP associated with viral infections have increased, further research is needed to fully comprehend the relationship between the two. This case aims to emphasize the importance of differentiating AEP from pneumonia caused by COVID-19 and promptly identifying it to enhance patient prognosis.
